# Draft Genome Sequence of *Necropsobacter rosorum* Strain P709^T^

**DOI:** 10.1128/genomeA.00913-14

**Published:** 2014-10-09

**Authors:** Roshan Padmanabhan, Catherine Robert, Florence Fenollar, Didier Raoult, Pierre-Edouard Fournier

**Affiliations:** a Aix-Marseille Université, URMITE, UM63, CNRS 7278, IRD 198, INSERM U1095, Faculté de Médecine, Marseille, France; b Special Infectious Agents Unit, King Fahd Medical Research Center, King Abdulaziz University, Jeddah, Saudi Arabia

## Abstract

*Necropsobacter* is a recently described genus that contains a single species, *N. rosorum*, and belongs to the family *Pasteurellaceae*. Here, we present the draft genome of *N. rosorum* strain P709^T^, which is the first genome sequence from this species.

## GENOME ANNOUNCEMENT

The genus *Necropsobacter* was first described in 2011 by Christensen et al. (1) on the basis of the phenotypic and genotypic characterization of 30 animal isolates (rodents, rabbits, dog) and 5 human isolates. The monophyly of the new genus, *Necropsobacter* gen. nov., was clearly demonstrated by the analysis of the 16S rRNA, *rpoB*, and *recN* genes. The type species of the genus, *N. rosorum*, was also first described by Christensen et al. (1). These bacteria are facultative anaerobes, Gram negative, coccoid or pleomorphic rods, nonmotile, nonhemolytic, and nonpigmented.

These bacteria are potentially infectious for humans. Several cases of bacteremia and/or focal suppuration have been reported (1–4). However, it is likely that the pathogenic role of *N. rosorum* is underestimated, as it is consistently misidentified by current commercial identification systems. This also explains the difficulty of accurate characterization of diseases caused by this organism and highlights 16S rRNA PCR and sequencing as the only reliable means to identify it.

Genome sequencing was performed using the MiSeq sequencer (Illumina, San Diego, CA) with a 2× 250-bp paired-end run after library preparation with the Nextera Xt sample preparation kit (Illumina). *De novo* assembly was done using CLC Genomics Workbench v7.0.4, and the resulting 48 contigs (>200 bp and >20× coverage with an *N*_50_ of 216,921) were used for open reading frame (ORF) prediction and gene annotation, carried out using the Prodigal software in the Prokka annotation suite (http://www.vicbioinformatics.com/software.prokka.shtml). RNAmmer (5) and tRNAscan-SE (6) were used to identify rRNA and tRNAs. Signal peptides were identified using SignalP v4.1 (7). The clustered regularly interspaced short palindromic repeats (CRISPR) were identified using CRISPRFinder (8).

The draft genome sequence of *Necropsobacter rosorum* is made of 48 chromosomal contigs exhibiting an average length and coverage of 52,618.5 bp and 223×, respectively. The draft genome length is 2,525,687 bp with a G+C content of 48.9%. The chromosome contains 2,301 protein-encoding genes and 7 rRNAs (1 rRNA contig and 4 additional 5S rRNAs), 51 tRNAs, 1 transfer-messenger (tmRNA), 27 other RNAs, and 188 signal peptides. Of the 2,301 genes, 2,146 (93.2%) and 1,850 (80.3%) were assigned a putative function by comparison to the Clusters of Orthologous Groups (COG) and KEGG databases, respectively. In addition, 155 genes (6.7%) encoded proteins classified as hypothetical. One CRISPR array was detected. Two intact (79.9-kb and 26.1-kb) and one questionable (6-kb) phage regions were identified in the genome using PHAST (9) with a total of 133 phage related genes. Using PathogenFinder, a Web server for the prediction of bacterial pathogenicity (10), three positive hits for pathogenic genes predicted a potential for human pathogenicity.

### Nucleotide sequence accession numbers.

This whole-genome shotgun project has been deposited at DDBJ/EMBL/GenBank under the accession numbers CCMQ01000001 to CCMQ01000048.
